# Phloem differentiation: an integrative model for cell specification

**DOI:** 10.1007/s10265-017-0999-0

**Published:** 2017-12-04

**Authors:** Bernhard Blob, Jung-ok Heo, Yka Helariutta

**Affiliations:** 1 Sainsbury Laboratory, Cambridge University, Bateman Street, Cambridge, CB2 1LR UK; 2 Institute of Biotechnology, University of Helsinki, 00014 Helsinki, Finland

**Keywords:** Auxin, Phloem sieve elements, Plethora, Single-cell transcriptomics

## Abstract

Plant vasculature consists of two major conductive cell types, xylem tracheary elements and phloem sieve elements (SEs). Both cell types undergo a highly specialized differentiation process. The root meristem of *Arabidopsis* displays a stereotypical anatomy in which the central vasculature is surrounded by concentric layers of outer tissues. Each cell file is derived from stem cells located in the root tip. A series of formative and proliferative divisions take place in the meristem; these are followed by cell expansion and differentiation. Protophloem differentiation is unique in being complete only 20–25 cells away from the first stem cell, and during the differentiation process the cells lose several organelles, including the nucleus, while the remaining organelles are rearranged. Defects in SE development have been shown to result in impaired auxin transport and response and therefore systemically affect root growth. Although a few genes have been demonstrated to function in phloem development, detailed analyses and a comprehensive understanding of sieve element development (i.e. how often the stem cells divide, how frequently enucleation takes place, and how SE development is coordinated between cell division and differentiation on a molecular level) are still lacking. Advanced live-imaging techniques which enable prolonged time-lapse captures of root tip growth as well as single-cell transcriptomic analysis of the 20–25 cells in the SE file could help resolve these questions. In addition, understanding the interplay between the PLETHORA (PLT) gradient, which is known to govern the root zonation, and phloem development within the root meristem could shed light on the rapidity of SE differentiation and its importance to the meristem.

## Introduction

In the vascular tissue of plants, the conductive cells in phloem and xylem are responsible for long distance transport. The sieve elements (SE) in the phloem are the main cells transporting photosynthates from source tissues, such as leaves, towards the parts of the plant where they are consumed, also referred to as sink tissues. The SEs consist of elongated cells with a modified and strengthened cell wall that are connected via sieve plates, specialized cell walls that have many large perforations to enable pressure-driven mass flow (Knoblauch et al. 2016). These cells undergo a special differentiation program that culminates in enucleation and clearing of most of the cellular content to reduce flow resistance yet remain alive (De Rybel et al. 2016; Furuta et al. 2014; Heo et al. 2017).

## The ontogeny of phloem sieve element along the longitudinal axis of the *Arabidopsis* root

**Fig. 1:**
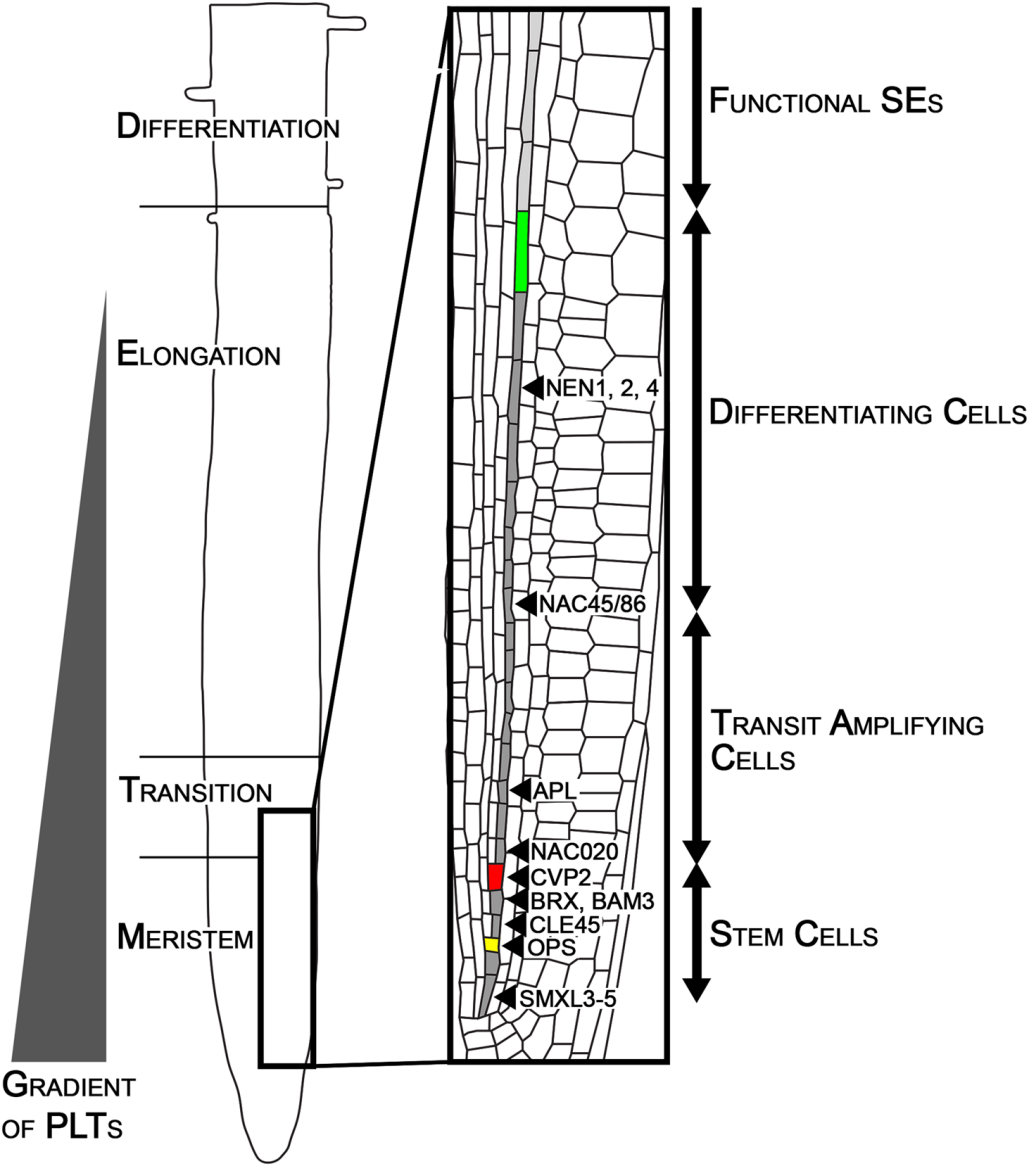
Overview of protophloem SE differentiation within the root meristem of *Arabidopsis thaliana*. 4 zones in the root tip can be distinguished: Meristem, Transition, Elongation and Differentiation zone (Ivanov and Dubrovsky 2013). The gradient of protein abundance of the PLTs is shown on the left side (Mähönen et al. 2014). The protophloem SE cell file is fully differentiated before the end of the transition zone as shown in the enlargement. The timing of differentiation progress has not been investigated yet, thus indicated zones within the SE cell stand are approximations. The first cell of the expression domains of known SE regulators is indicated with arrow heads. The yellow and red cell mark the first and second periclinal cell division, respectively. The transit amplifying cells divide several times anticlinally. The differentiating cells elongate and undergo cell wall thickening, sieve plate morphogenesis and autolysis. The green cell is enucleating

In the root of *Arabidopsis thaliana*, the central vascular cylinder is surrounded by concentric layers of tissues, displaying a stereotypical anatomy. Tight regulation of the timing of cell proliferation and the differentiation of each tissue layer is necessary for continuous apical growth of the root (Beemster and Baskin 1998). The root meristem resides at the root tip and consists of the stem cell niche, a collective term for the mitotically less active quiescent center and the surrounding stem cells, and transit amplifying cells (Dolan et al. 1993). Upon exiting from the cell division zone, cells begin to expand and become committed to a particular cell fate, a process which results in a longitudinal zonation pattern (Fig. 1; Ivanov and Dubrovsky 2013). The switch from cell division to elongation and differentiation occurs at slightly different points for each cell type (Ishikawa and Evans 1995). For instance, stem cells in the phloem SE lineages divide anticlinally to produce SE/procambium precursor cells which then undergo a periclinal cell division to give rise to SE precursors and procambial cells (Mähönen et al. 2000). SE precursors undergo yet another periclinalcell division, producing two different types of SEs, proto- and meta-phloem SEs. After this series of periclinal divisions, the cells enter the proliferation stage where they undergo several yet uncharacterized anticlinal divisions before expanding and differentiating (Fig. 1; Mähönen et al. 2014). SEs undergo a specialized differentiation process, during which the cellular components are partially degraded and rearranged within a stretch of approximately 10 cells before enucleation (Furuta et al. 2014; Heo et al. 2017). From the stem cell touching the quiescent centre (QC) to the cell which is about to lose its nucleus, there are approximately 20–25 cells. After losing the nucleus, protophloem SEs immediately become part of the ‘phloem unloading zone’, facilitating the translocation of small molecules, such as sucrose or GFP, towards the cortex layer through the neighboring pericycle cells and subsequently to the entire root meristem (Ross-Elliott et al. 2017). SEs are unique in that their differentiation is complete within 20–25 cells, at which point the neighboring cells are still dividing. Five days after germination, the cortex file consists of approximately 30 dividing cells in the meristem before elongation begins, and differentiated protoxylem can only be observed at much later stages, when root hair outgrowth from the epidermis occurs (Dello Ioio et al. 2007; Truernit et al. 2012). The stochasticity in the initiation of differentiation suggests that a cell autonomous (intrinsic) mechanism governs the establishment and maintenance of specific cell lineages, and non-cell-autonomous signals mediate cell-to-cell communication to coordinate root development and growth.

## Molecular mechanism regulating sieve element specification and differentiation

The molecular mechanisms regulating the differentiation of phloem cells are still largely unknown, although some progress has been made in unravelling them in recent years. The subclade 2 of the SUPRESSOR OF MAX2-LIKE (SMXL) genes, namely SMXL3, SMXL4 and SMXL5, play redundantly a major role in early protophloem development. Although not exclusively expressed in the phloem, their expression in root meristem starts from the first stem cell adjacent to the QC. The combinatorial double mutants of these three genes all show strongly reduced root growth while the *smxl3 smxl4 smxl5* tripple mutant is seedling lethal. In the more detailed analysis of *smxl4 smxl5* revealed alterations or lack of distinct cellular changes of protophloem cells, e.g. enucleation does not occur resulting in reduced phloem sap transport (Wallner et al. 2017). Another gene that acts very early is *OCTOPUS* (OPS). The OPS protein is polarly localized on the shootward plasma membrane (Truernit et al. 2012). It can interact with and sequesters BRASSINOSTEROID INSENSITIVE2, a repressor of the brassinosteroid signalling pathway, to the plasma membrane, thus lifting the repression (Anne et al. 2015). However, this interaction and the shootward plasma membrane localization appear not to be essential for the function of OPS in protophloem differentiation, since both are lost in plants with a hyperactive OPS protein resulting from a positive charge at a phosphosite without causing defects in phloem development (Breda et al. 2017). In the *ops* knock-out mutant protophloem cells do not fully differentiate; cell wall thickening cannot be observed, sieve plates are not formed and the nucleus is retained, resulting in a reduced translocation rate of phloem sap (Truernit et al. 2012). These cells interrupt and form gaps within the SE strand continuity, thus these cells were dubbed gap cells (Breda et al. 2017).

The small mobile protein CLAVATA3/EMBRYO SURROUNDING REGION 45 (CLE45) is the next factor known to be involved in SE differentiation; it is expressed one cell later than OPS. CLE45 is perceived by the receptor BARELY ANY MERISTEM 3 (BAM3), and its signalling is further enhanced by MEMBRANE-ASSOCIATED KINASE REGULATOR 5 and CORYNE (CRN) (Depuydt et al. 2013; Hazak et al. 2017; Kang and Hardtke 2016; Rodriguez-Villalon et al. 2014). Exogenous application of CLE45 locks the protophloem SEs in their pre-differentiation state, and overexpression of a modified, less active version of the peptide inhibits differentiation of some cells in the cell file, resulting in a gap cell phenotype similar to that of *ops* (Kang and Hardtke 2016; Rodriguez-Villalon et al. 2014). In addition, other CLE peptides affect root growth although the protophloem specific functions, receptors and their cross-talk are unknown (Hazak et al. 2017). CLE26 is the best investigated one, which is expressed in a protophloem specific manner towards the end of differentiation and similar to CLE45 requires CRN to be perceived (Hazak et al. 2017; Rodriguez-Villalon et al. 2015).

*BREVIS RADIX* (*BRX*), a putative repressor of the CLE45/BAM3 signalling pathway, mediates auxin-brassinosteroid signalling cross talk in the root meristem. Its knock-out mutant also shows the gap cell phenotype. Interestingly, the phenotypes of *brx* and *ops* are additive, suggesting that these genes have parallel functions in similar processes (Breda et al. 2017; Kang and Hardtke 2016; Scacchi et al. 2009), although the full effect and interplay of these components is not fully understood.

A gap cell phenotype can also be seen following perturbation of the phosphatidylinositol-4,5-biphosphate (PtdIns(4,5)P_2_) level in developing protophloem SEs of the double mutant of the phosphoinositide 5-phosphatases *COTELYDON VASCULAR PATTERN 2* (*CVP2*) and *CVP2-LIKE 1*. Mutations of *CVP2* can rescue *brx* but not *ops* mutant. Yet increased OPS dosage can rescue the *cvp2 cvl1* mutant, placing *OPS* downstream of PtdIns(4,5)P_2_ levels (Rodriguez-Villalon et al. 2015). At a lower level of penetrance, the same phenotype is also seen in the combinatorial mutant of *BRASSINOSTEROID INSENSITIVE 1* (*BRI1*), *BRI1-LIKE1* (*BRL1*) and *BRL3* (Kang et al. 2017). Although severe growth phenotypes have sometimes been observed in these mutants and overexpressors, phloem development was never completely abolished, suggesting the existence of other important factors that have not yet been elucidated. In addition, the second periclinal cell division in the phloem lineage does not occur in *ops, brx*, and the CLE45 overexpressor. It has been suggested that this is a secondary effect of a top-down signal being inhibited because of the gap cells, but these findings could also hint at further regulatory mechanisms active before the onset of SE differentiation (Rodriguez-Villalon et al. 2014).

Interestingly, in the recently developed Vascular Cell Induction Culture System Using *Arabidopsis* Leaves (VISUAL) in which partial SE differentiation can be induced in leaf tissue, cell divisions are an essential step for SE differentiation. Work using this culture system identified *NAC DOMAIN-CONTAINING PROTEIN 20* (*NAC020*) as an important regulator of SE differentiation. It is thought to act as a repressor genetically upstream of *ALTERED PHLOEM* (*APL*), though results from in vitro and in planta experiments are partially contradictory regarding its mode of activity (Kondo et al. 2016). *APL* was one of the earliest described key regulators of phloem development. It is necessary for the differentiation of functional SEs and represses xylem identity in the position where protophloem SEs form (Bonke et al. 2003). In recent years, other NAC-type transcription factors, *NAC86* and *NAC45*, have been identified as downstream targets of *APL*. They coordinate the expression of *NAC45*/*86-DEPENDENT EXONUCLEASE-DOMAIN PROTEIN* (*NEN*) *1, 2* and *4* and the translocation of *NEN1* and *NEN2* from the cytosol to the nucleus upon enucleation. Both the NACs and their downstream targets the NENs are essential for full degradation of the nucleus (Furuta et al. 2014). However, molecular features of SEs, such as sieve plate formation, can still be found in cells in the SE position in *apl* mutant roots, suggesting that unknown regulatory elements coordinate essential events other than enucleation late during SE differentiation (Truernit et al. 2008). For example, *CHOLINE TRANSPORTER-LIKE1* plays an important role in the formation of sieve plate pores, although this is likely due to its more general function in plasmodesmata development (Dettmer et al. 2014; Kraner et al. 2017).

All of the regulatory mechanisms described so far are specific to the SEs or phloem; the role of general regulators or stem cell activity and cell division has not been taken into account, and very little is known about their specific role in SE differentiation.

## Phloem as a hub for systemic communication within the root meristem

The primary transport sugar sucrose and the plant hormone auxin have been shown to be transported over long distances through the phloem and to play roles as signaling molecules that can change the expression of genes (Bishopp et al. 2011; Turgeon and Oparka 2010; Yoo et al. 2004, 2013). Therefore, defects in phloem development can have a systemic adverse impact on the root meristem and root growth in general. In fact, mutations in genes important for phloem development often result in short root phenotypes in addition to altered SE differentiation.

In addition to gap cells, *ops* displays defects in cell division and elongation during embryogenesis—cells divide in a position where they normally undergo elongation (Truernit et al. 2012). Long-distance transport is impaired, and auxin transport from fully differentiated SEs to immature SEs was therefore thought to be affected as well, leading to a secondary phenotype in the meristem in which the division that gives rise to meta-/proto-phloem sieve elements occurs at a lower frequency (Rodriguez-Villalon et al. 2014). The increase of number of lateral roots observed in this mutant could be explained by the accumulation of auxin at higher regions due to the impaired long distance transport. Similar phenotypes have been reported in *brx, cvp2 cvl1*, and CLE45-treated roots (Depuydt et al. 2013; Hazak et al. 2017; Rodriguez-Villalon et al. 2015; Scacchi et al. 2010).

More direct experimental evidence also supports the requirement for auxin in SE development. Upon auxin treatment, *BRX* promoter activity is induced and the BRX protein becomes dissociated from the plasma membrane and accumulates in the nucleus, where it appears to be subject to auxin-induced degradation (Scacchi et al. 2009). Although these observations were made in other cells, it is likely that they also occur in SEs as suggested by mathematical models (Santuari et al. 2011). Furthermore, BRX is thought to be asymmetrically localized in the basal end of the cell similar to the auxin transporter PIN-FORMED1 and full-length BRX driven by the *PIN1* promoter can fully rescue the *brx* short root phenotype (Mouchel et al. 2006; Scacchi et al. 2009). BRX appears to directly interact with the auxin response factor MONOPTEROS. This supports the notion that BRX at the plasma membrane could act as a messenger to convey the auxin signal into the nucleus, where initiation of downstream gene expression takes place (Scacchi et al. 2010, 2009). Interestingly, *brx* roots are non-responsive to cytokinin treatment, highlighting the importance of *brx* and the cross-regulation of these two hormones (Scacchi et al. 2010).The *apl* mutant and the *nac45*/*nac86* double mutant also display impaired root growth, though it remains unclear whether the impaired root growth phenotype is due to disturbed auxin transport or response, or to the lack of carbohydrate availability (Bonke et al. 2003; Furuta et al. 2014).

The *PLETHORA* (*PLT*) genes are transcription factors that are downstream of auxin (Aida et al. 2004). They form a gradient in the root meristem, with high PLT levels in meristematic cells adjacent to the QC, medium levels in transit amplifying cells, and low levels in differentiating cells (Galinha et al. 2007). The distribution of the PLT protein results from a transcriptional gradient and cell-to-cell movement of the protein and was shown to be responsible for the longitudinal zonation of the root. High concentrations of the PLTs slow down cell division in the stem cell niche, whereas medium levels activate mitotic cell division. Introduction of an extra copy of PLT leads to a shootward shift in the expression of the mitotic cell division marker *CYCB1;1*. In addition, ectopic expression of *PLT2* in the protoxylem and epidermal cells represses differentiation locally, indicating that the PLTs repress differentiation. The decrease in PLT levels along the gradient is therefore able to determine where the transition to differentiation occurs. Although PLTs are downstream of auxin, they appear to act in parallel with auxin in the formation of root zones, as their expression does not change immediately after auxin treatment or in the *aux1 ein2 gnom* mutant background (Mähönen et al. 2014).

However, tissue-specific integration of the auxin and PLT signals has not yet been discovered in the vasculature of the root. Each tissue layer differentiates at its own pace, raising the question of how each cell layer perceives the auxin and PLT signals and how individual tissue types communicate with each other to maintain the root meristem and achieve balanced root growth (Ubeda-Tomás et al. 2012). One possibility is provided by a recent analysis indicating that the PLT genes not only activate various genes involved in the cell proliferative function of the meristem but also repress genes that promote cell differentiation (Santuari et al. 2016). In the future, it will be important to accurately establish the PLT expression domain during phloem development.

## Concluding remarks and future perspective

Visualization techniques have continued to improve in recent years, opening up new avenues for the study of plant development. These include the further development and use of light sheet fluorescence microscopy for in vivo observations and the use of serial block phase scanning electron microscopy for 3D reconstructions at an extremely detailed level (Furuta et al. 2014; Ovečka et al. 2015). Collectively, these new techniques will help answer questions that could not be addressed previously (e.g. How high is the rate of division in cells of the stem cell niche? How often does enucleation take place? How long does it take for a cell to progress through the full SE differentiation program from the stem cell touching the QC to enucleated cells?). Recent advances in RNA-sequencing and single cell transcriptomics will also help to elucidate these processes at the level of a single cell. While single-cell RNA-sequencing has already been used for several years with animal cells, in plants it can be combined with established protoplasting and FACS methods to offer enormous potential for understanding the developmental programs of the root (Efroni et al. 2016; Efroni and Birnbaum 2016). These approaches will doubtlessly have many applications, including the identification of novel phloem genes and attaining a comprehensive understanding of phloem development at the molecular/gene expression level. This could serve as a basis for computational models of regulatory networks which could then be verified by classical reverse genetics approaches (Balaguer and Sozzani 2017).

## References

[CR1] Aida M, Beis D, Heidstra R, Willemsen V, Blilou I, Galinha C, Nussaume L, Noh Y-S, Amasino R, Scheres B (2004). The PLETHORA genes mediate patterning of the *Arabidopsis* root stem cell niche. Cell.

[CR2] Anne P, Azzopardi M, Gissot L, Beaubiat S, Hématy K, Palauqui J-C (2015). OCTOPUS negatively regulates BIN2 to control phloem differentiation in *Arabidopsis thaliana*. Curr Biol.

[CR3] Balaguer MAdeLuis, Sozzani R (2017). Inferring gene regulatory networks in the *Arabidopsis* root using a dynamic Bayesian network approach. Methods Mol Biol.

[CR4] Beemster GTS, Baskin TI (1998). Analysis of cell division and elongation underlying the developmental acceleration of root growth in *Arabidopsis thaliana*. Plant Physiol.

[CR5] Bishopp A, Help H, El-Showk S, Weijers D, Scheres B, Friml J, Benková E, Mähönen AP, Helariutta Y (2011). A mutually inhibitory interaction between auxin and cytokinin specifies vascular pattern in roots. Curr Biol.

[CR6] Bonke M, Thitamadee S, Mähönen AP, Hauser M-T, Helariutta Y (2003). APL regulates vascular tissue identity in *Arabidopsis*. Nature.

[CR7] Breda AS, Hazak O, Hardtke CS (2017). Phosphosite charge rather than shootward localization determines OCTOPUS activity in root protophloem. Proc Natl Acad Sci.

[CR8] De Rybel B, Mähönen AP, Helariutta Y, Weijers D (2016). Plant vascular development: from early specification to differentiation. Nat Rev Mol Cell Biol.

[CR9] Dello Ioio R, Linhares FS, Scacchi E, Casamitjana-Martinez E, Heidstra R, Costantino P, Sabatini S (2007). Cytokinins determine *Arabidopsis* root-meristem size by controlling cell differentiation. Curr Biol.

[CR10] Depuydt S, Rodriguez-Villalon A, Santuari L, Wyser-Rmili C, Ragni L, Hardtke CS (2013). Suppression of *Arabidopsis* protophloem differentiation and root meristem growth by CLE45 requires the receptor-like kinase BAM3. Proc Natl Acad Sci.

[CR11] Dettmer J, Ursache R, Campilho A, Miyashima S, Belevich I, O’Regan S, Mullendore DL, Yadav SR, Lanz C, Beverina L, Papagni A, Schneeberger K, Weigel D, Stierhof Y-D, Moritz T, Knoblauch M, Jokitalo E, Helariutta Y (2014.) CHOLINE TRANSPORTER-LIKE1 is required for sieve plate development to mediate long-distance cell-to-cell communication. Nat Commun 5:4276. 10.1038/ncomms5276 10.1038/ncomms5276 25008948

[CR12] Dolan L, Janmaat K, Willemsen V, Linstead P, Poethig S, Roberts K, Scheres B (1993). Cellular organisation of the *Arabidopsis thaliana* root. Development.

[CR13] Efroni I, Birnbaum KD (2016). The potential of single-cell profiling in plants. Genome Biol.

[CR14] Efroni I, Mello A, Nawy T, Ip P-L, Rahni R, DelRose N, Powers A, Satija R, Birnbaum KD (2016). Root regeneration triggers an embryo-like sequence guided by hormonal interactions. Cell.

[CR15] Furuta KM, Yadav SR, Lehesranta S, Belevich I, Miyashima S, Heo J, Vatén A, Lindgren O, Rybel BD, Isterdael GV, Somervuo P, Lichtenberger R, Rocha R, Thitamadee S, Tähtiharju S, Auvinen P, Beeckman T, Jokitalo E, Helariutta Y (2014). *Arabidopsis* NAC45/86 direct sieve element morphogenesis culminating in enucleation. Science.

[CR16] Galinha C, Hofhuis H, Luijten M, Willemsen V, Blilou I, Heidstra R, Scheres B (2007). PLETHORA proteins as dose-dependent master regulators of *Arabidopsis* root development. Nature.

[CR17] Hazak O, Brandt B, Cattaneo P, Santiago J, Rodriguez-Villalon A, Hothorn M, Hardtke CS (2017). Perception of root-active CLE peptides requires CORYNE function in the phloem vasculature. EMBO Rep.

[CR18] Heo J-O, Blob B, Helariutta Y (2017). Differentiation of conductive cells: a matter of life and death. Curr Opin Plant Biol.

[CR19] Ishikawa H, Evans ML (1995). Specialized zones of development in roots. Plant Physiol.

[CR20] Ivanov VB, Dubrovsky JG (2013). Longitudinal zonation pattern in plant roots: conflicts and solutions. Trends Plant Sci.

[CR21] Kang YH, Hardtke CS (2016). *Arabidopsis* MAKR5 is a positive effector of BAM3-dependent CLE45 signaling. EMBO Rep.

[CR22] Kang YH, Breda A, Hardtke CS (2017). Brassinosteroid signaling directs formative cell divisions and protophloem differentiation in *Arabidopsis* root meristems. Development.

[CR23] Knoblauch M, Knoblauch J, Mullendore DL, Savage JA, Babst BA, Beecher SD, Dodgen AC, Jensen KH, Holbrook NM (2016). Testing the Münch hypothesis of long distance phloem transport in plants. eLife.

[CR24] Kondo Y, Nurani AM, Saito C, Ichihashi Y, Saito M, Yamazaki K, Mitsuda N, Ohme-Takagi M, Fukuda H (2016). Vascular cell induction culture system using *Arabidopsis* leaves (VISUAL) reveals the sequential differentiation of sieve element-like cells. Plant Cell.

[CR25] Kraner ME, Link K, Melzer M, Ekici AB, Uebe S, Tarazona P, Feussner I, Hofmann J, Sonnewald U (2017). Choline transporter-like1 (CHER1) is crucial for plasmodesmata maturation in *Arabidopsis thaliana*. Plant J.

[CR26] Mähönen AP, Bonke M, Kauppinen L, Riikonen M, Benfey PN, Helariutta Y (2000). A novel two-component hybrid molecule regulates vascular morphogenesis of the *Arabidopsis* root. Genes Dev.

[CR27] Mähönen AP, ten Tusscher K, Siligato R, Smetana O, Díaz-Triviño S, Salojärvi J, Wachsman G, Prasad K, Heidstra R, Scheres B (2014). PLETHORA gradient formation mechanism separates auxin responses. Nature.

[CR28] Mouchel CF, Osmont KS, Hardtke CS (2006). BRX mediates feedback between brassinosteroid levels and auxin signalling in root growth. Nature.

[CR29] Ovečka M, Vaškebová L, Komis G, Luptovčiak I, Smertenko A, Šamaj J (2015). Preparation of plants for developmental and cellular imaging by light-sheet microscopy. Nat Protoc.

[CR30] Rodriguez-Villalon A, Gujas B, Kang YH, Breda AS, Cattaneo P, Depuydt S, Hardtke CS (2014). Molecular genetic framework for protophloem formation. Proc Natl Acad Sci.

[CR31] Rodriguez-Villalon A, Gujas B, van Wijk R, Munnik T, Hardtke CS (2015). Primary root protophloem differentiation requires balanced phosphatidylinositol-4,5-biphosphate levels and systemically affects root branching. Dev Camb Engl.

[CR32] Ross-Elliott TJ, Jensen KH, Haaning KS, Wager BM, Knoblauch J, Howell AH, Mullendore DL, Monteith AG, Paultre D, Yan D, Otero S, Bourdon M, Sager R, Lee J-Y, Helariutta Y, Knoblauch M, Oparka KJ (2017). Phloem unloading in *Arabidopsis* roots is convective and regulated by the phloem-pole pericycle. eLife.

[CR33] Santuari L, Scacchi E, Rodriguez-Villalon A, Salinas P, Dohmann EMN, Brunoud G, Vernoux T, Smith RS, Hardtke CS (2011). Positional information by differential endocytosis splits auxin response to drive *Arabidopsis* root meristem growth. Curr Biol.

[CR34] Santuari L, Sanchez-Perez GF, Luijten M, Rutjens B, Terpstra I, Berke L, Gorte M, Prasad K, Bao D, Timmermans-Hereijgers JLPM, Maeo K, Nakamura K, Shimotohno A, Pencik A, Novak O, Ljung K, van Heesch S, de Bruijn E, Cuppen E, Willemsen V, Mähönen AP, Lukowitz W, Snel B, de Ridder D, Scheres B, Heidstra R (2016). The PLETHORA gene regulatory network guides growth and cell differentiation in *Arabidopsis* roots. Plant Cell.

[CR35] Scacchi E, Osmont KS, Beuchat J, Salinas P, Navarrete-Gómez M, Trigueros M, Ferrándiz C, Hardtke CS (2009). Dynamic, auxin-responsive plasma membrane-to-nucleus movement of *Arabidopsis* BRX. Dev Camb Engl.

[CR36] Scacchi E, Salinas P, Gujas B, Santuari L, Krogan N, Ragni L, Berleth T, Hardtke CS (2010). Spatio-temporal sequence of cross-regulatory events in root meristem growth. Proc Natl Acad Sci USA.

[CR37] Truernit E, Bauby H, Dubreucq B, Grandjean O, Runions J, Barthélémy J, Palauqui J-C (2008). High-resolution whole-mount imaging of three-dimensional tissue organization and gene expression enables the study of Phloem development and structure in *Arabidopsis*. Plant Cell.

[CR38] Truernit E, Bauby H, Belcram K, Barthélémy J, Palauqui J-C (2012). OCTOPUS, a polarly localised membrane-associated protein, regulates phloem differentiation entry in *Arabidopsis thaliana*. Dev Camb Engl.

[CR39] Turgeon R, Oparka K (2010). The secret phloem of pumpkins. Proc Natl Acad Sci.

[CR40] Ubeda-Tomás S, Beemster GTS, Bennett MJ (2012). Hormonal regulation of root growth: integrating local activities into global behaviour. Trends Plant Sci.

[CR41] Wallner E-S, López-Salmerón V, Belevich I, Poschet G, Jung I, Grünwald K, Sevilem I, Jokitalo E, Hell R, Helariutta Y, Agustí J, Lebovka I, Greb T (2017). Strigolactone- and karrikin-independent SMXL proteins are central regulators of phloem formation. Curr Biol.

[CR42] Yoo B-C, Kragler F, Varkonyi-Gasic E, Haywood V, Archer-Evans S, Lee YM, Lough TJ, Lucas WJ (2004). A systemic small RNA Ssignaling system in plants. Plant Cell.

[CR43] Yoo S-C, Chen C, Rojas M, Daimon Y, Ham B-K, Araki T, Lucas WJ (2013). Phloem long-distance delivery of FLOWERING LOCUS T (FT) to the apex. Plant J Cell Mol Biol.

